# Total laparoscopic hysterectomy versus abdominal hysterectomy in the treatment of patients with early stage endometrial cancer: A randomized multi center study

**DOI:** 10.1186/1471-2407-9-23

**Published:** 2009-01-15

**Authors:** Claudia BM Bijen, Justine M Briët, Geertruida H de Bock, Henriëtte JG Arts, Johanna A Bergsma-Kadijk, Marian JE Mourits

**Affiliations:** 1 Department of Gynecology, University Medical Center Groningen, University of Groningen, Groningen, The Netherlands; 2 Department of Epidemiology, University Medical Center Groningen, University of Groningen, Groningen The Netherlands

## Abstract

**Background:**

Traditionally standard treatment for patients with early stage endometrial cancer (EC) is total abdominal hysterectomy and bilateral salpingo oophorectomy (TAH+BSO) with or without lymph node dissection through a vertical midline incision. While TAH is an accepted effective treatment, it is highly invasive, visibly scarring and associated with morbidity. An alternative treatment is the same operation by laparoscopy. Though in several studies total laparoscopic hysterectomy (TLH+ BSO) seems a safe and feasible alternative approach in early stage endometrial cancer patients, there are no randomized data available yet. Furthermore, a randomized controlled trial with surgeons trained in laparoscopy is warranted in order to implement this technique in a safe manner. The aim of this study is to compare the treatment related morbidity, cost-effectiveness and quality of life in early stage endometrial cancer patients treated by laparoscopy versus the standard open approach.

**Methods:**

A multi centre randomized clinical phase 3 trial, including 5 university hospitals and 15 regional hospitals in the Netherlands. Only gynecologists trained in performing a TLH are allowed to participate. Inclusion criteria: Patients with a clinical stage I endometrioid adenocarcinoma or complex atypical hyperplasia are randomized in a 2:1 allocation to receive TLH or TAH. The main outcome measure is the rate of major complications, as assessed by an independent clinical review board. In total, 275 patients are required to have 80% power at α-0.05 to detect a significant difference of 15% complication rate. Secondary outcome measures are 1) costs and cost-effectiveness, 2) minor complications, and 3) quality of life. All data from this multi center study are reported using case record forms. Data regarding quality of life, pain, body Image, sexuality and additional homecare are assessed with self reported questionnaires.

**Discussion:**

A randomized multi center study in early stage endometrial cancer patients with inclusion criteria for patients and surgeons is designed and ongoing. Results will be presented at the end of 2009.

**Trial Registration:**

*Dutch trial register number NTR821*.

## Background

Endometrial cancer is the third most common cancer in women in Western countries, accounting for 6–9% of their cancers, with a peak incidence at the age of 55–65 year. Endometrial cancer is a disease of the elderly and 90% of patients are over 50 years of age. The incidence increases in obese women and 70% of the patients have a high body mass index (BMI >25) and 50% have co-morbidity such as diabetes and cardiovascular disease. A total of 75% of the patients are diagnosed with stage I disease. Traditionally standard treatment for patients with early stage endometrial cancer (EC) is total abdominal hysterectomy and bilateral salpingo oophorectomy (TAH+BSO) with or without lymph node dissection through a vertical midline incision. While TAH is an accepted effective treatment, it is highly invasive, visibly scarring and associated with adverse events such as blood loss and wound problems [1,2]. Morbidity of laparotomy in case of endometrial cancer can be substantial due to frequent obesity and co-morbidity in this patient group and hospital stay is usually at least one week [1]. A good alternative approach for patients with early stage disease is by laparoscopy.

Laparoscopy in early stage endometrial cancer is a minimal invasive technique compared to the standard approach by laparotomy. In several retrospective and prospective studies it has been shown that the laparoscopic approach is an effective and safe alternative to the open procedure. Most of these studies show a significant reduction in treatment related morbidity, with shorter hospital stay, less pain and quicker return to activities in daily life with the laparoscopic approach compared to laparotomy [1-10]. The advantages of the laparoscopic approach seem to be even more pronounced in obese [2] and elderly patients [10,11] due to reduced complications and a shorter hospital stay. However, the only randomized controlled trial comparing the laparoscopic approach with the open approach was in benign disease [12]. This study is in a different group of patients (young, healthy) than the study population of the here proposed study i.e. elderly women with endometrial cancer. It is known that complications occur more frequently during the initial learning curve of this procedure and some authors suggest a quantity control (i.e. > 25 TLH procedures) for surgeons before participating in studies. [12]

Arguments often mentioned hampering the implementation of level three laparoscopic procedures are the high preoperative costs and the long learning curve. However, despite the fact that peroperative costs are higher for laparoscopy due to expensive disposables, it might be that the overall costs will be finely balanced between both procedures due to reduction of morbidity and shorter hospital stay [13,14].

Although randomized studies on this issue are not available yet, studies in which endometrial cancer patients are treated show similar rates and patterns of recurrence. Patients who underwent a TLH+BSO have similar recurrence rates when compared to TAH+BSO, as reported in a retrospective mono-centre study [3] and in two prospective mono-center studies [6,15]. In view of these data we proposed a randomized controlled clinical trial in which total laparoscopic hysterectomy performed by trained surgeons is compared to total abdominal hysterectomy in patients with early stage endometrial cancer.

## Methods

### Aims&design

The aim of this study is to compare treatment related morbidity, cost-effectiveness and quality of life in early stage endometrial cancer treated by laparoscopy (TLH+BSO) or laparotomy (TAH+BSO).

We hypothesize that laparoscopy will result in less treatment-related morbidity in patients treated for early stage endometrial cancer in a cost-effective manner. The study will also provide insight on whether the laparoscopic approach will improve quality of life as compared to the standard abdominal approach. The proposed research concerns a multi-center prospective randomized clinical phase 3 trial (including at least 15 centers) comparing major complication rate in patients with early stage endometrial cancer, randomized to surgical treatment by laparotomy (TAH+BSO) or laparoscopy (TLH+BSO).

### Eligibility criteria

#### Inclusion criteria patients

Early stage endometrial cancer patients (endometrioid adenocarcinoma grade 1 or 2, clinically stage I disease, negative endocervical curettage), after signed written informed consent, age 18 years and older are eligible for this trial.

#### Exclusion criteria patients

Exclusion criteria: other histological types than endometrioid adenocarcinoma of the endometrium, clinically advanced disease (stage II to IV), uterine size larger than conform 10 weeks gestation and cardio pulmonary contra indications for laparoscopy.

### Patient recruitment and consent

Eligible patients are identified and counseled by the gynecological staff of participating hospitals. Before entry into the study the gynecologist explains to potential subjects the aims, methods, reasonably anticipated benefits and potential hazards of the study. Before study entry, on all patients an endocervical curettage is performed to make sure that the patient has clinical stage I disease and the cervix is not involved.

Subjects are informed that their participation is voluntary and that they may withdraw consent to participate at any time during the study. In every center an independent medical doctor is available for more detailed information both for patients and colleagues if required. After sufficient information, written informed consent has to be obtained. The consent form must be signed before performance of any study-related activity. Patients who decide not to participate in this study are treated according to standard treatment protocol (TAH+BSO). Patients undergo routine physical examination and blood tests prior to the operation in either treatment arm, which is part of the standard procedure before surgery.

### Randomization and collection of baseline data

**Figure 1:**
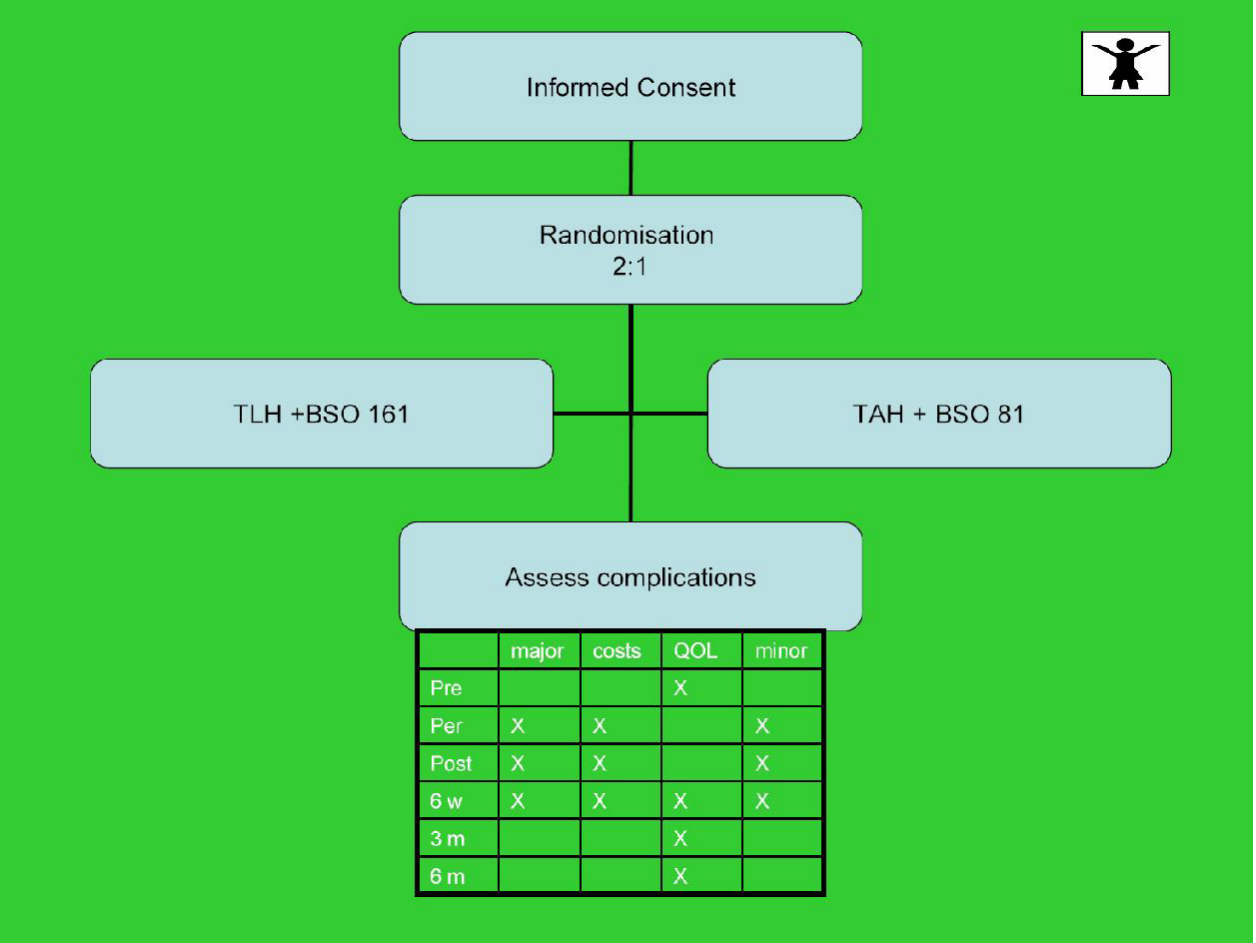
**Assessment schedule**.

The patients to be enrolled in this trial are allocated to the TAH or TLH arm by computer randomization. For randomization, blocks of x patients are created such that balance is enforced within each block, stratified per center. Randomization is 2:1 for intervention to provide more exposure to data on the new laparoscopic procedure. We also expect a better enrolment in the study if the patients have a twofold chance of being allocated to the laparoscopic group as the reduced morbidity seems to be substantial. At study entry, all woman have baseline demographic, past gynecologic and medical history recorded in a case record form (CRF). After randomization, but prior to surgical treatment, patients are asked to fill in a questionnaire, consisting items regarding: quality of life (SF-36, EQ-5D), pain (VAS), Body Image Scale (BIS), sexuality (SAQ) and a questionnaire on additional homecare. (see figure 1)

At local centers, data collection is the responsibility of the local participating gynecologist and research nurses. The data collected in this study are coded and processed with adequate precautions to ensure patient confidentiality.

### Interventions

In this trial, the standard surgical approach TAH +BSO (table 1) is compared to the investigative surgical procedure TLH+BSO (table 2).

**Table 1:** Surgical treatment protocol for a TAH, total abdominal hysterectomy

Preoperatively thrombosis prophylaxis is given
Preoperative antibiotic is given at least 15 min before skin incision;

Positioning of the patient in lithotomy position.

Vertical midline incision

Abdominal washing for cytology

Bipolar coagulation or sealing of the round ligament, cutting with monopolar scissors. Opening of the peritoneum of the bladder and the pelvic sidewall.

Bipolar coagulation or sealing of the infundibulopelvic ligament, cutting with monopolar scissors

Preparation of the bladder off the vagina

Skeletting the uterine vessels, coagulation or sealing of the vessels, after identification of the ureter.

Coagulation or sealing and cutting of the sacrouterine ligaments.

Taking out the uterus. Closing of the vaginal cuff by abdominal stitching.

Mass closure of sheath, skin closure.

**Table 2:** Surgical treatment protocol for a TLH, total laparoscopic hysterectomy

Preoperatively thrombosis prophylaxis is given
Preoperative antibiotic is given at least 15 min before skin incision;

Positioning of the patient in lithotomy position.

Insufflation of CO2 and placing of the troicarts (4).

Abdominal washing for cytology.

Bipolar coagulation or sealing of the round ligament, cutting with monopolar scissors. Opening of the peritoneum of the bladder and the pelvic sidewall.

Bipolar coagulation or sealing of the infundibulopelvic ligament, cutting with monopolar scissors.

Placing the vaginal tube (the Mc Cartney tube). Preparation of the bladder off the vagina.

Skeletting the uterine vessels, coagulation or sealing of the vessels, after identification of the ureter.

Coagulation or sealing and cutting of the sacrouterine ligaments.

Cutting the vaginal wall on the rim of the vaginal tube. Keeping the ureter in sight.

Taking out the uterus. Closing of the vaginal cuff by abdominal or vaginal stitching.

### Follow-up

Outcomes are assessed pre-operatively, after 6 weeks, and after 3 and 6 months, being the moments of routine control assessments and be registered on a case record form (CRF) by the treating physician and checked by the research nurse (status review) (see figure 1). Costs are assessed by a cost assessment (CRF and patient questionnaire). The doctor registers costs on a CRF related to the use of operation materials (disposables), operating time, duration of hospital stay, and treatment of complications.

All adverse events are followed until they have abated, or until a stable situation has been reached.

Data regarding quality of life (SF-36, EQ-5D), pain (VAS), Body Image Scale (BIS), sexuality (SAQ) and additional homecare are assessed with help of self reported questionnaires 6 weeks, 3 and 6 months after the operation (see figure 1).

### Monitoring

#### Pre start monitoring

To minimize complications due to inexperience, all inexperienced general gynecologists are trained by a visiting gynecologist with experience in laparoscopy until sufficient laparoscopic skills are reached, before study entry. Only gynecologists who reached sufficient scores (≥ 28 points) on OSATS in performing two independent TLH procedures are allowed to participate in the study. The study coordinator facilitates centers in order to get approval of the medical ethical committee. As the study is set in a national research consortium, allied research nurses are responsible for collecting data, monitoring of the study and promote the study at the local centers.

A kick off meeting is held by the study coordinator in every participating center. During this meeting the content and inclusion procedure of the study is explained.

#### On site monitoring

During the study period, frequent contact and assistance of the coordination center is enabled by regular telephone calls and visits to all local centers. Furthermore, every three months newsletters by email are send to the centers in order to inform the gynecologists about study progress and bring new items under their attention.

Publicity of the study is realized by means of presentations held on national and international congresses. Thereby, the study protocol and other related study documents are available online. *http://www.studies-obsgyn.nl/home/page.asp?page_id=418*.

Case record forms, checked by the research nurse of concerned center and self reported patient questionnaires are send to the Trial Coordination Center (TCC) of the coordinating center. The TCC takes care of entry and validating data of case record forms and importing patient questionnaires. In case of discrepancies or inaccuracies found in the case record forms, queries are send to the centers in question. Final monitoring will be done by the study coordinator by visiting randomly selected centers and checking a part of the case record forms once again.

### Study parameters/endpoints

#### Main study parameter/endpoint

Our primary endpoint is: major complication rate. The following major complications are registered: injuries of bowel, bladder, ureter, vessel, nerves; thrombo-embolic events such as DVT (Deep Venous Thrombosis) or pulmonary embolism; haematoma requiring surgical intervention; hemorrhage requiring transfusion and/or surgical intervention; wound dehiscence requiring surgical intervention or re-admission; wound infections including vaginal vault abscess, requiring surgical intervention and/or prolonged hospital stay and/or readmission and/or treatment; other major complications.

The severity of a complication is scored according to the Common Terminology Criteria CTCAE version 3.0. An independent Complication Review Board of three experienced clinicians is asked to assess and judge all recorded complications and differentiate between major and minor complications, blinded to treatment arm. This Complication Review Board decides also if the complication is related to the operative procedure, as expressed by the following statements: not related, unlikely related, possibly related, probably related or definitely related. *http://ctep.cancer.gov/protocolDevelopment/electronic_applications/docs/newadverse_2006.pdf*

#### Secondary study parameters/endpoints

1) Cost effectiveness

2) Minor complications. Outcomes are assessed by analyzing the CRF. An independent clinical review panel differentiates between major and minor complications, blinded for treatment arm and also assess whether the complication is related to the operative procedure.

3) Quality of life (SF-36 and EUROQOL), sexual functioning (Sexual Activity Questionnaire), Body Image Scale (BIS) and pain (Visual Analogue Scale).

### Premature termination of the study

The study will be terminated prematurely if the disadvantages of participation may be significantly greater than was foreseen in the research proposal. All complications are assessed and recorded by an independent clinical review panel. This panel assesses if the complications occurring in the TLH group do exceed in amount or severity the complications occurring in the standard of care (TAH) group. If so, the study will be terminated.

### Statistical analysis

#### Sample size

Sample size estimates are based on the ability of the study to detect changes in the primary outcome measure, rate of major complications. In this study, we choose for an unbalanced randomization (2(TLH+BSO):1(TAH+BSO)). Group sample sizes of 161 and 81 achieve 80% power to detect a difference of 0.15 between the null hypothesis that the major complication rate is 25% in both the TAH + BSO and the TLH + BSO group and the alternative hypothesis that we will observe a complication rate of 25% in the TAH + BSO group and of 10% in the TLH + BSO group, using a two-sided Chi-square test with continuity correction and with a significance level of 0.05. Assuming a drop-out percentage of 10%, 275 patients are needed.

The assumptions regarding these expected complication rates are based on the complication rates reported in (retrospective) studies comparing TAH with TLH. In addition, this is supported by the major complication rate of 9.26% that we found during the learning curve in our pilot study. The drop-out percentage of 10% can be expected when patients, after randomization, are unhappy with their allocated treatment and choose to end their participation in the study or due to incomplete or lost data.

#### Descriptive statistics

Primary endpoints will be analyzed according to the intention-to-treat principle.

Descriptive statistics for QOL (SF-36, EQ-5D, VAS, BIS, SAQ) and a questionnaire on additional homecare) will be calculated for each randomization group at each assessment. Similarly, descriptive statistics will also be calculated for other outcomes, such as pain scores, and analgesic consumption, etc. Continuous variables will be assessed for normality and equality of variances between groups. Discrete variables (e.g. presence/absence of post-operative infection) will be summarized by frequencies/proportions. For continuous variables, analysis of variance and/or regression will be used, where appropriate. If assumptions for these tests are violated, alternative non-parametric tests will be used. Difference between groups with respect to discrete variables will be evaluated by using chi-squared tests.

#### Economic analysis

In the economic evaluation the costs of both interventions will be compared. The economic evaluation will be conducted from a societal perspective including direct medical and direct non-medical costs. Relevant cost components that will be taken into account are costs of the laparotomy and laparoscopy, like costs of operating time and use of disposables. In addition, hospital stay, re-interventions for post-operative morbidity and operation-related medication will be assessed. In case of conversion from TLH to TAH the costs for both procedures will be added to the costs in the TLH group. Home care, consisting of both professional care as well as informal care will be assessed as well. Because most women are aged 55 and older, productivity losses will not be included in the economic evaluation.

Cost components will be valued according to standard Dutch guidelines for economic evaluation (CVZ 2004). Actual costs will be estimated for the laparotomy and laparoscopy and informal care will be valued by using shadow prices.

A case record form will be used to gather medical costs. A patient questionnaire will be used to collect information on additional home care. Since no differences regarding recurrence rate are expected at any time between the two study arms, a time horizon of six months is considered to be sufficient for evaluation of morbidity and costs. As a consequence, discounting will not be applied. A sensitivity analysis will be conducted to estimate the impact of variation of major cost elements.

### Ethics

The study is conducted according to the principles of the Declaration of Helsinki and in accordance with the Medical Research Involving Human Subjects Act (WMO).

The protocol is registered in the clinical Dutch trial register number NTR821.

## Discussion and conclusion

While the standard abdominal hysterectomy for early stage endometrial cancer is an effective and accepted treatment in patients with early stage endometrial cancer, it is highly invasive and associated with serious adverse events. From retrospective or non-randomized trials can be concluded that laparoscopic hysterectomy seems a safe and feasible treatment especially in patients with early stage endometrial cancer.

GOG-LAP2, a randomized controlled trial in which the effectiveness of a laparoscopic assisted vaginal hysterectomy with BSO and lymphadenectomy in early stage endometrial cancer is compared to the open procedure, has been completed. A major problem in the GOG-LAP2 study is that inexperienced gynecologists in laparoscopic surgery are allowed to participate and no quality control for the laparoscopists or the laparoscopic procedure is performed. Recently, a randomized multi center trial comparing the laparoscopic with the open approach in early stage endometrial cancer called the LACE trial, has been started in Australia. These studies however, are not applicable to the European situation, because in these studies patients are mostly treated by a hysterectomy and BSO combined with a pelvic and para aortic lymphadenectomy. In Europe, a lymphadenectomy is not standard treatment for patients with early stage endometrial cancer. Moreover, early stage endometrial cancer patients in our proposed study only undergo a hysterectomy with BSO and most of these procedures are performed in general hospitals by general gynecologists.

Therefore, a randomized multi center trial with on-site monitoring containing inclusion criteria for patients as well as for surgeons is designed to provide evidence on short term outcome between laparoscopy and laparotomy in patients with early stage endometrial cancer. Results will be presented at the end of 2009.

## Abbreviations

BIS: Body Image Scale; BSO: Bilateral Salpingo Oophorectomy; CRF: Case Record Form; CTCAE: Common Terminology Criteria of Adverse Events; DVT: Deep Venous Thrombosis; EC: Endometrial Cancer; EQ-5D: EuroQol-5 dimensional; OSATS: Objective Structured Assessment of Technical Skills; SAQ: Sexual Activity Questionnaire; SF-36: Short Form-36; TAH: Total Abdominal Hysterectomy; TCC: Trial Coordination Center; TLH: Total Laparoscopic Hysterectomy; VAS: Visual Analogue Scale; WMO: Wet Medisch Wetenschappelijk Onderzoek.

## Competing interests

The authors declare that they have no competing interests.

## Authors' contributions

GdB, JBK, JB, HA and MM were involved in conception and design of the study based on their preclinical and clinical results and experiences. GdB formulated the statistical analysis plan. GdB, MM and JB drafted the manuscript. CB coordinates the study and wrote the manuscript. JBK coordinates the entry and validation of data. All authors mentioned in the manuscript are members of the TLH/TAH study committee. All authors read and approved the final manuscript.

## Pre-publication history

The pre-publication history for this paper can be accessed here:

http://www.biomedcentral.com/1471-2407/9/23/prepub
